# Understanding the impact of symptoms on the burden of COPD

**DOI:** 10.1186/s12931-017-0548-3

**Published:** 2017-04-21

**Authors:** Marc Miravitlles, Anna Ribera

**Affiliations:** 1 Pneumology Department, Vall d’Hebron University Hospital, CIBER de Enfermedades Respiratorias (CIBERES), P. de la Vall d’Hebron 119-129, 08035 Barcelona, Spain; 2 AstraZeneca PLC, Avda. Diagonal 615, 08028 Barcelona, Spain

**Keywords:** Chronic obstructive pulmonary disease, Symptoms, Burden, Variability, Patient-reported outcomes

## Abstract

Chronic obstructive pulmonary disease (COPD) imposes a substantial burden on individuals with the disease, which can include a range of symptoms (breathlessness, cough, sputum production, wheeze, chest tightness) of varying severities. We present an overview of the biomedical literature describing reported relationships between COPD symptoms and disease burden in terms of quality of life, health status, daily activities, physical activity, sleep, comorbid anxiety, and depression, as well as risk of exacerbations and disease prognosis. In addition, the substantial variability of COPD symptoms encountered (morning, daytime, and nighttime) is addressed and their implications for disease burden considered. The findings from this narrative review, which mainly focuses on real-world and observational studies, demonstrate the impact of COPD symptoms on the burden of disease and that improved recognition and understanding of their impact is central to alleviating this burden.

## Background

Chronic obstructive pulmonary disease (COPD) is associated with a significant socio-economic burden, which is predicted to increase over the coming decades [1, 2]. A range of symptoms and their impact on patients define the daily burden of COPD borne by an individual. The most common symptoms of COPD are dyspnea, cough, and sputum production, and less common but troublesome symptoms are wheezing, chest tightness, and chest congestion. However, reported frequencies differ depending on the patient population and severity of disease [3]. For example, cough has been reported as the most common symptom in patients with mild COPD [4].

The majority of individuals with COPD perceive symptom burden as a significant ongoing challenge to performing their day-to-day activities. For example, in a European, cross-sectional observational study investigating perceptions of symptoms and their impact on daily life activities among patients with COPD (*n* = 2441), 92.5% of patients reported experiencing ≥1 COPD symptom during the previous week [5]. Moreover, 33–50% of patients indicated that their COPD symptoms affected them the most during every day of the previous week.

The importance of symptoms in COPD is acknowledged by the current Global initiative for chronic Obstructive Lung Disease (GOLD) document, which recommends evaluating symptom burden (primarily dyspnea) and exacerbation history separately from airflow limitation. While spirometric measurements are required to make a diagnosis of COPD, the evaluation of respiratory symptoms is crucial for the therapeutic decision. The report also acknowledges that the most common respiratory symptoms, including dyspnea, cough and/or sputum production may be under-reported by patients [6]. Of note, the use of spirometry alone has under-served physicians in terms of understanding the adverse effects of COPD on patient health-related quality of life; however, this shortcoming can be addressed by the routine use of validated and reliable questionnaires assessing COPD symptoms and daily functioning [7]. Moreover, it is the symptoms of COPD or an exacerbation, rather than airflow limitation, that initially motivates patients to seek professional medical help [8, 9].

Given the increased recognition of COPD symptoms as a key component of the GOLD combined disease assessment approach, their role in precipitating interactions between patients and healthcare professionals, and reports of patients’ perceptions of COPD and its individualized impact on their lives, this narrative review provides an overview of the biomedical literature describing, and the evidence base supporting, the importance of symptoms in driving COPD burden. The review encompasses data from real-world experience and clinical trials addressing the variability of COPD symptoms, the relationship between COPD symptoms and quality of life, the impact of COPD symptom burden in terms of patients’ day-to-day and physical activities, the relationship between COPD symptoms and mood abnormalities (anxiety/depression), the impact of COPD symptoms on sleep, and the relationship between symptom burden risk of exacerbations and disease prognosis.

The articles included in this narrative review were selected if they reported the measurement of symptoms of COPD in terms of variability, frequency, and overall burden, or if they reported the association of COPD symptoms in relation to quality of life, health status, daily activities, physical activity, sleep, comorbid anxiety and depression, exacerbations, and disease prognosis. PubMed was used to identify manuscripts of interest based on appropriate search terms (for example, ‘copd [Title/Abstract] AND symptoms [Title/Abstract] AND sleep [Title/Abstract]’) and the resulting articles were subsequently selected for relevance.

## The burden and high variability of COPD symptoms

The perception of COPD as an unremitting, progressive disease with increasing levels of symptoms associated with worsening lung function and characterized by limited variability in symptom presentation has been refuted by an increasing evidence base and improved understanding of the disease. Beyond the now established poor correlation between symptom perception and forced expiratory volume in 1 s (FEV_1_), it is now acknowledged that COPD symptoms show high seasonal, weekly, and daily variability [10]. Breathlessness is the hallmark symptom of COPD and there is an increasing evidence base demonstrating that the overall symptomatic burden (which may also include cough, sputum production, wheeze, and chest tightness) has a substantial detrimental impact on health status, quality of life, and daily activities, and also contributes to increased anxiety and depression levels, increased risk of exacerbations, and a worse disease prognosis [6, 11–16].

Patients have reported that the morning is the worst time of day for symptoms of COPD, with cough and sputum production being most troublesome [17, 18]. The need to ameliorate COPD morning symptoms is reinforced by their association with poorer health status, reduction in daily living activities, and increased exacerbation risk [18]. The presence of morning symptoms of COPD have also been shown to have a negative impact on daytime physical activity [19].

Nighttime symptoms and sleep disturbance are prevalent yet under-recognized in patients with COPD, and there is a paucity of clinical research into COPD nighttime symptoms [20, 21]. This situation is particularly worrisome given the potential detrimental clinical impact of COPD nighttime symptoms and sleep disturbance on long-term changes in lung function, exacerbation frequency, cardiovascular disease risk, cognition, depression, quality of life, and increased mortality [20, 21]. Sleep disturbance in COPD is discussed in more detail later.

In a study by Kessler et al., the majority of symptomatic patients (62.7%) self-reported perceptions of variability in at least one COPD symptom [5]. Daily, weekly, and seasonal variability in their COPD symptoms was reported by 44.7%, 54.4%, and 59.5% of patients, respectively. Breathlessness was most commonly cited as the symptom that showed variability on both a daily and weekly basis. Notably, greater variability in patient-reported breathlessness across the week was associated with an increased detrimental impact on daily activities throughout the 24-h day. Of the patients who reported seasonal variability in their COPD symptoms, 55.9% believed that their symptom burden was greatest during the winter months. This latter observation is consistent with data from the TORCH study, which showed that winter is associated with an increased risk of COPD exacerbations and the hypothesis that the cold, damp environment prevailing during the winter months, as well as increased exposure to the influenza virus at this time of year, may partly explain this seasonal association [22, 23].

Lung function shows circadian variation even in healthy individuals, so it is perhaps unsurprising that many patients with COPD experience variation in their symptoms over the course of the day, with the most severe symptoms occurring during the early morning and nighttime [5, 14, 17, 20, 24].

ASSESS was a pan-European, non-interventional, observational study that recorded the prevalence and severity of symptoms in patients with stable COPD (*n* = 727) throughout the 24-h day (early morning, daytime, and nighttime) and investigated their effects on a broad range of patient-reported outcomes [3]. The results showed that despite receiving regular treatment, 90.5% of patients experienced COPD symptoms during any part of the day and 56.7% had symptoms during each part of the day [3]. Among patients who reported experiencing ≥1 symptom in the previous week, symptoms were more frequently encountered during the early morning (81.4%) and daytime (82.7%) periods than at nighttime; however, nighttime symptoms were also very common (63.0%). Furthermore, >80% of patients in each disease severity category (based on airflow limitation) reported having COPD symptoms (84.1% in ‘mild’, 88.7% in ‘moderate’, 93.9% in ‘severe’, and 91.8% in ‘very severe’). Even those patients diagnosed with mild COPD reported experiencing symptoms in all three parts of the day (early morning, 44.1%; daytime, 43.1%; nighttime, 46.7%). A significant relationship between increasing COPD severity and higher prevalence of symptoms during early morning and daytime was identified (*p* < 0.05, each); however, no such trend was evident for nighttime symptoms. An association was also observed between early-morning and daytime symptoms at baseline and the presence of exacerbations during the 6-month follow-up period (both *p* < 0.01) [25]. This suggests that there may be a relationship between 24-h COPD symptoms and the frequency of exacerbations.

**Fig. 1:**
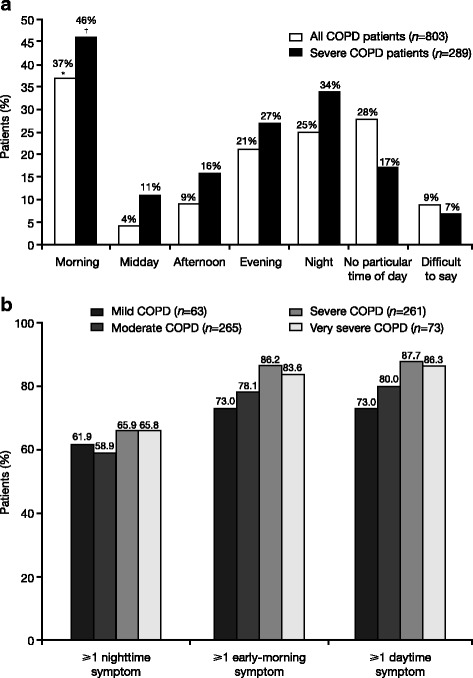
**a** Time of day when COPD symptoms are worse than usual. Reproduced from [17]; **b** prevalence of any COPD symptoms during each part of the 24-h day, according to COPD severity. Reproduced from [3]. *p < 0.001 vs ‘midday’, ‘afternoon’, ‘evening’, ‘night’, and ‘difficult to say’ groups; *p* = 0.006 vs ‘no particular time of day’ (all COPD patients); ^†^ *p* < 0.001 vs ‘midday’. *COPD* chronic obstructive pulmonary disease

The diurnal variation in COPD symptoms described in the ASSESS study is consistent with other published studies that have reported the variability in COPD symptoms during different parts of the 24-h day [5, 10, 13, 14, 17, 18, 26–28]. However, there is also considerable variability in the prevalence of symptoms during each part of the day reported by different studies (Table 1). A cross-sectional survey of 1489 patients with COPD found that 39.8% of patients experienced early-morning symptoms, 97% experienced daytime symptoms, and 58% experienced nighttime symptoms [18]. Another cross-sectional survey of 1239 patients reported that 61.2% of patients experienced both early-morning and nighttime symptoms of COPD, whilst 17.4% reported early-morning symptoms only and 4.8% reported nighttime symptoms only [27]. Furthermore, an internet survey of 803 patients with COPD found that 37% of patients reported experiencing worse symptoms in the morning and 25% of patients reported nighttime as the worst time of day for symptoms, whilst this percentage increased in those patients with severe COPD [17] (Fig. 1). A pooled analysis of 3394 patients with moderate to severe COPD participating in two large multinational Phase III clinical trials reported that 94.4% of patients reported experiencing early-morning symptoms at baseline and 88.3% of patients reported experiencing nighttime symptoms at baseline [29]. Symptoms of COPD occurring in the early morning, daytime, or at nighttime can have a serious impact on a patient’s daily living activities and quality of life and this is discussed in more detail in the next section.

**Table 1:** Variability of COPD symptom prevalence in different studies

Study	Patients	Symptoms	Prevalence, %		
			Morning	Daytime	Nighttime
Miravitlles et al. COPD 2016 [25]	*n* = 727	Any symptoms	81.4	82.7	63.0
Stephenson et al. Int J Chron Obstruct Pulmon Dis. 2015 [27]	*n* = 1239	Any symptoms	78.6	n.r.	65.9
Bateman et al. Respir Res. 2015 [29]	*n* = 3394	Any symptoms	94.4	n.r.	88.3
Roche et al. COPD 2013 [18]	*n* = 1489	Any symptoms	39.8^a^	97	58
Partridge et al. Curr Med Res Opin. 2009 [17]	*n* = 803	Worse symptoms^b^	37	34	25

^a^Morning symptoms were defined as those symptoms present on waking, rather than those persisting through the morning

^b^Defined as symptoms that were worse than usual

*COPD* chronic obstructive pulmonary disease, *n.r.* not reported

## Impact of COPD symptoms on quality of life

Real-world data suggest that patients with COPD who experience early-morning and nighttime symptoms are significantly more likely to have worse health-related quality of life than those without [13, 27]. Furthermore, 1-year follow-up data from a multicenter, prospective study of patients with COPD (*n* = 791) showed that deterioration in health-related quality of life was associated with significant increases in COPD respiratory symptoms (dyspnea, coughing, and expectoration) [12]. Survey data from patients with COPD (*n* = 1100) identified increased coughing, shortness of breath, fatigue, and increased sputum production as the exacerbation symptoms that had the greatest impact on their wellbeing (42%, 37%, 37%, and 35%, respectively) [11].

Findings from the ASSESS study showed that overall health status, as defined by the COPD Assessment Test (CAT), was significantly lower in the cohort of patients who had at least one COPD symptom versus the cohort that reported no COPD symptoms, and this trend was observed consistently in the early morning, daytime, and nighttime (*p* < 0.001, each). Moreover, the magnitude of differences in CAT scores between cohorts was greater than the two-point minimum clinically important difference on this outcome measure, thereby implicating higher symptomatic burden with a clinically relevant detrimental impact on patients’ overall health status. Of note, the presence of ≥1 COPD symptom was also associated with significantly higher levels of dyspnea in each part of the day [3].

Real-world data from the European, cross-sectional, observational HEED study of patients with COPD (*n* = 2294) demonstrated that dyspnea was more common in patients with ‘severe’ (91.6%) or ‘very severe’ (99.2%) primary care physician-rated COPD than in those with ‘mild’ (42.2%) or ‘moderate’ (69.8%) disease [4]. Dyspnea grade, primary care physician-rated COPD severity, sputum production, and number of comorbidities were identified as significant factors associated with health status as measured by St George’s Respiratory Questionnaire (SGRQ) and CAT scores (*p* < 0.0001, all). These findings are consistent with those from the ASSESS study, demonstrating that even patients with mild COPD encounter a significant self-reported symptom burden [3].

Given the increasing recognition of, and importance attributed to, patients’ views and preferences on their disease and its management, it is important to identify and understand differences between patients’ and physicians’ perception of COPD symptoms. An observational, cross-sectional descriptive study identified concordance between patients with moderate or severe COPD (*n* = 450) and their pulmonologists (*n* = 77) in terms of ‘breathlessness/shortness of breath’, ‘fatigue/tiredness’, and ‘coughing’ being the most relevant symptoms [30]. These findings were based on concordance between the overall groups; however, subsequent analysis between each patient and their corresponding physician identified poor concordance, with only 53% agreeing which symptom most concerned or affected the life of the patient. Concordance was greater between physicians and patients who had more severe COPD compared with those who had moderate disease, which may be attributable to higher exacerbation/hospitalization risk, higher frequency/intensity of COPD symptoms, and improved patient-physician communication.

## COPD symptoms and physical activity

Physical activity is consistently associated with clinical and functional determinants of COPD (including dyspnea, quality of life, and exercise capacity) [31], with symptoms found to have a significant negative impact on patients’ level of physical activity, irrespective of time of day [3]. Patients with COPD perceive that symptoms can impose a substantial limitation on their ability to perform normal activities throughout the 24-h day (including physical activity and exercise) and can impair sleep quality [5]. Morning symptoms of COPD are considered by patients to be a key barrier to performing their daily activities [5, 10, 17, 27, 28]. Furthermore, morning symptoms are associated with a higher likelihood of workplace absenteeism [18]. A range of daily activities (e.g. ‘going up and down stairs’, ‘doing heavy household chores’, ‘going shopping’, and ‘taking part in sports and hobbies’) and morning-specific daily activities (‘washing’, ‘dressing’, ‘drying’, and ‘getting out of bed’) have been cited by patients as the aspects of their normal functioning most compromised by COPD symptoms, with some patients requiring assistance to successfully complete daily activities due to their level of impaired daily functioning, thus leading them to perceive themselves as a burden on others [5]. Patients with COPD start to reduce physical activity levels early in disease progression in order to avoid symptoms such as dyspnea [32, 33]. The resultant muscle deconditioning, which is present even in mild disease [34, 35], contributes further to a vicious cycle of inactivity [36, 37]. Maintaining physical activity levels is important in COPD as it is associated with a better disease prognosis, as well as reduced hospitalization and mortality [38–40]. The latest GOLD update acknowledges the potential of behavior-targeted interventions, and recommends motivating patients to do more physical activity [6]. While existing evidence is compelling, future research is required and the incorporation of physical activity outcome measures into randomized controlled trials is necessary.

## Impact of COPD symptoms on anxiety and depression

It has been noted that patients with COPD experience worse psychological functioning and greater psychological distress than patients with other chronic medical conditions, and that lack of mental health knowledge among healthcare workers may be a barrier to diagnosis and access to appropriate treatment interventions [41]. Anxiety and depression are important comorbidities in patients with COPD and their negative effects on mortality, exacerbation rates, length of hospital stay, quality of life, and functional status in patients with COPD are being increasingly recognized [42].

The cause-and-effect relationship between dyspnea and anxiety/depression is complicated by the overlap between the symptoms of COPD and those of anxiety [43], however there is evidence to suggest that there is a relationship between increased dyspnea and anxiety/depression in patients with COPD. Two observational, cross-sectional, multicenter studies investigating factors associated with depression and anxiety in COPD found that patients with depression had greater dyspnea that those without. In the DEPREPOC (Depression in Chronic Obstructive Pulmonary Disease) study of 836 patients (83% male; mean age, 68.3 years), depressive symptoms were measured using the Beck Depression Inventory questionnaire and the study found that the presence of depression in patients with COPD was associated with greater dyspnea as measured by the modified Medical Research Council dyspnea scale (depression, 2.07; no depression, 1.32; *p* < 0.0001) [44]. Furthermore, an observational study conducted in 115 patients with stable COPD found that patients with depression (measured by the Hospital Anxiety and Depression Scale [HADS]) showed greater dyspnea compared with patients without depression [45].

An analysis of data from a randomized controlled study of male patients with COPD (*n* = 162; mean age, 67.1 years) was conducted in order to evaluate the association between anxiety/depression and pulmonary-specific symptoms, and to investigate the potential moderating effects of disease severity and functional capacity on any relationship [16]. Anxiety and depression (as measured by the State-Trait Anxiety Inventory and Beck Depression Inventory, respectively) were each associated with higher levels of fatigue, shortness of breath, and frequency of COPD symptoms. Moreover, functional capacity (6-Min Walk Test) but not disease severity (FEV_1_) was identified as a significant moderator of anxiety and pulmonary-specific COPD symptoms. Specifically, the detrimental effects of anxiety on shortness of breath and COPD symptoms frequency observed in patients with anxiety were significantly greater among those with lower functional capacity.

Further evidence supporting the negative association between COPD symptom burden and depressed mood comes from a prospective cohort study of patients hospitalized due to an exacerbation of their COPD (*n* = 376; median follow-up, 369 days) [46]. Patients with comorbid depression at baseline (HADS score ≥8) experienced a significantly higher symptom burden, as measured by the SGRQ symptom subscale, than those without depression at index hospitalization (68.6 vs 60.3; *p* = 0.003) and 1-year follow-up (66.6 vs 56.5; *p* = 0.006), which accounted for a 12.1% and 15.2% increase in symptom burden, respectively.

The aforementioned ASSESS study evaluated the impact of COPD symptoms across the 24-h day on patient-reported outcomes, including levels of anxiety and depression [3]. Patients with ≥1 COPD symptom in each part of the 24-h day (nighttime, early morning, and daytime), experienced significantly higher levels of anxiety and depression (measured by HADS) compared with those who had no COPD symptoms (*p* < 0.001, each).

## Impact of COPD symptoms on sleep

**Fig. 2:**
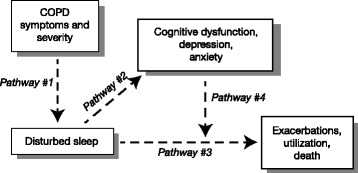
Cross-sectional associations (Pathways #1 and #2), longitudinal associations (Pathway #3), and cognitive deficits or psychological factors as potential mediators in longitudinal associations (Pathway #4) as described by Omachi et al. Reproduced from [52]. *COPD* chronic obstructive pulmonary disease

Sleep disturbances are common among patients with COPD, affecting in excess of 70% of patients [13, 47, 48]. Sleep disturbances include difficulties in initiating and maintaining sleep and increased number of arousals during the night, and arise from a combination of disturbances in ventilation and gas exchange caused by the underlying condition [49, 50] and disruption caused by nighttime respiratory symptoms (particularly coughing, breathlessness, and sputum production) and other generalized symptoms such as chest pain, heartburn/palpitations, and nighttime fear and anxiety [20, 51]. As previously noted, sleep quality is a major determinant of health-related quality of life in patients with COPD [47, 51], and sleep disturbances are associated with poor health outcomes [52]. Sleep-related breathing disturbances in patients with COPD result in hypoxemia and hypercapnia, which are associated with cardiac arrhythmias, pulmonary hypertension, and nocturnal death, especially during acute exacerbations [50]. Disturbed sleep leads to difficulties in getting up in the morning [13] and is associated with depressive and anxiety symptoms [51, 52]. Sleep disturbances have been shown to be greater in patients with worse dyspnea upon exertion and are also associated with reduced subsequent daytime physical activity [53]. In a European retrospective analysis of real-world data describing 2807 patients with COPD, 78% of patients experienced physician-reported nighttime disturbances due to symptoms including ‘trouble falling asleep’, ‘wake up several times per night’, ‘trouble staying asleep’, and ‘wake up feeling tired and worn-out after usual amount of sleep’ [13]. The researchers identified a higher incidence of daytime breathlessness and more frequent exacerbations within the previous 12 months in patients who experienced COPD nighttime symptoms than in those who had no nighttime symptoms. Furthermore, the presence of nighttime symptoms was associated with a greater likelihood of experiencing COPD morning symptoms, disturbed sleep, and poorer quality of life [13]. These findings were consistent with other reports that nighttime symptoms impair sleep quality and morning routine, which combine to compromise overall health status [5, 10, 17]. In ASSESS, the presence of ≥1 COPD symptom in any part of the day was associated with significantly worse sleep quality, and moreover patients with morning, daytime, or nighttime symptoms had significantly higher COPD and Asthma Sleep Impact Scale scores versus those without symptoms, indicating that these patients have greater sleep impairment [3]. A population-based, longitudinal study of 98 adults with COPD found that respiratory symptoms such as cough and breathlessness may be responsible for poor sleep quality; however, sleep disturbance was predictive of COPD exacerbations (odds ratio [OR] 4.7; 95% confidence interval [CI] 1.3, 17; *p* = 0.018), respiratory-related emergency healthcare utilization (OR 11.5; 95% CI 2.1, 62; *p* = 0.004), and all-cause mortality (hazard ratio [HR] 5.0; 95% CI 1.4, 18; *p* = 0.013), suggesting that sleep plays an independent role as a risk factor for worsening COPD and poor outcomes [52]. These cross-sectional and longitudinal associations are illustrated in Fig. 2.

## Impact of COPD symptoms on risk of exacerbations and disease prognosis

The presence of COPD symptoms at any time of day or night has been associated with a worse disease prognosis. An analysis of pooled data from two independent studies involving >6000 patients with COPD found that the presence of nighttime breathlessness was associated with future exacerbations (HR 2.3; 95% CI 1.7, 3.0), hospital admissions due to COPD (HR 3.2; 95% CI 2.3, 4.4), and mortality (HR 1.7; 95% CI 1.2, 2.3) [21]. Additionally, the ASSESS study found that patients who had early-morning or daytime symptoms at baseline had significantly more exacerbations during the following 6 months (*p* < 0.01); however, significance was not maintained when adjusted for potential confounding factors such as lung function [25]. Furthermore, a prospective, multicenter study of 227 patients identified a significant correlation between the level of breathlessness and 5-year mortality [54]. Chronic cough is a very common and troublesome symptom in patients with COPD and the presence of a productive cough may be indicative of progressive disease [55]. In addition, patients with a productive cough have been found to have an increased risk of exacerbations, hospitalization, and mortality [56–58].

## Conclusions

**Fig. 3:**
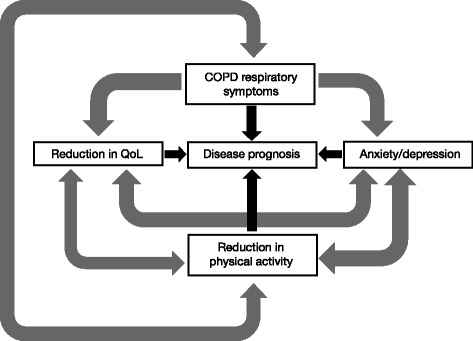
The relationship between dyspnea, depression/anxiety, reduction in physical activity, impact on quality of life, and disease prognosis. *COPD* chronic obstructive pulmonary disease; QoL, quality of life

COPD symptoms are associated with a clinically meaningful decline in the quality of life, overall health status, and prognosis of individuals with this disease (Table 2 and Fig. 3). COPD symptoms progressively compromise the patient’s ability to function normally in terms of their day-to-day activities and physical activity, and can impair sleep quality. Moreover, increased COPD symptom burden is associated with comorbid anxiety and depression. Furthermore, the presence of COPD symptoms is associated with an increased risk of exacerbations and a worse disease prognosis. The substantial variability of COPD symptoms experienced throughout the 24-h day (morning, daytime, and nighttime) and the poor concordance between physicians and patients in terms of COPD symptom impact and importance are a further important complication and challenge. Collectively, the evidence presented supports the important role of COPD symptoms in driving the burden of disease that is borne by the individual, and symptoms are therefore a key target in the treatment of COPD, which is in line with the recent GOLD update. Symptoms should be assessed routinely using patient-centered questionnaires and healthcare professionals should consider symptoms in the long-term treatment plan of patients with COPD as it is essential that they are managed effectively throughout the 24-h day.

**Table 2:** Studies investigating associations between COPD symptoms and other factors

Study	Measures	Association(s)
***Quality of life***		
Miravitlles et al. Respir Med. 2007 [11]	Patient questionnaire (daily wellbeing and COPD symptoms)	Increased coughing, followed by increasing shortness of breath, fatigue, and increased production of sputum were reported as having a strong impact on wellbeing
Jones et al. Prim Care Respir J. 2012 [4]	SGRQ (quality of life) CAT (quality of life) MRC scale (dyspnea) Patient questionnaire (symptoms)	Dyspnea grade, PCP-rated COPD severity, sputum production and number of comorbidities were significantly associated with SGRQ and CAT score (all *p* < 0.0001)
Price et al. Int J Chron Obstruct Pulmon Dis. 2013 [13]	EQ-5D (quality of life) Physician record (COPD symptoms time of day)	Patients with physician-reported nighttime symptoms had significantly poorer quality of life (*p* < 0.0001)
Monteagudo et al. Respir Med. 2013 [12]	SGRQ (quality of life) Patient interview (chronic respiratory symptoms)	Cough and sputum and increased dyspnea were associated with a significant worsening of HRQoL (all *p* < 0.001)
Miravitlles et al. Respir Res. 2014 [3]	CAT score (quality of life) Patient questionnaire (COPD symptoms)	Overall health status was significantly lower in patients with at least one symptom in the morning, daytime, or nighttime (*p* < 0.001)
Stephenson et al. Int J Chron Obstruct Pulmon Dis. 2015 [27]	CAT score (quality of life) Patient questionnaire (COPD symptoms)	Patients with both nighttime and early-morning symptoms were more likely to have poorer health status (OR 8.03; 95% CI 4.33, 14.89)
***Physical activity***		
Partridge et al. Curr Med Res Opin. 2009 [17]	Patient questionnaire (COPD symptoms)	The impact of COPD symptoms on morning activities is substantial, with dyspnea being the most problematic
Kessler et al. Eur Respir J. 2011 [5]	Patient questionnaire (COPD symptoms and impact on daily activities)	Morning symptoms of COPD had the greatest impact on daily living activities
O’Hagan and Chavannes. Curr Med Res Opin. 2014 [28]	Patient questionnaire (COPD symptoms and impact on daily activities)	With morning symptoms, routine activities took 10–15 min longer and more strenuous activities around 30 min longer
Stephenson et al. Int J Chron Obstruct Pulmon Dis. 2015 [27]	Patient questionnaire (COPD symptoms and limitation of activities)	60.4% of patients reported limiting their morning activity due to early-morning symptoms
Miravitlles et al. Respir Res. 2014 [3]	Patient questionnaire (COPD symptoms time of day and physical activity levels)	A higher proportion of patients who were sedentary had symptoms in the morning, daytime, and nighttime compared with active patients
***Depression***		
Ng et al. Arch Intern Med. 2007 [46]	HADS (depression) SGRQ (COPD symptom burden and QoL)	Increased symptom burden in patients with depression (*p* < 0.001)
Doyle et al. Int J Psychiatry Med. 2013 [16]	State-Trait Anxiety Inventory (anxiety) Beck Depression Inventory (depression) Brief Fatigue Inventory (fatigue) SGRQ (COPD symptoms) UCSD Shortness of Breath Questionnaire (dyspnea) 6MWT (functional capacity)	Anxiety and depression associated with higher fatigue, dyspnea, and frequency of COPD symptoms (all *p* < 0.001); more so in patients with lower functional capacity (*p* = 0.02–0.009)
Miravitlles et al. Respir Med. 2014 [44]	Beck Depression Inventory (depression) mMRC scale (dyspnea)	Greater dyspnea in patients with depression vs no depression (mean dyspnea grade: 2.07 vs 1.32; *p* < 0.0001)
Miravitlles et al. Respir Res. 2014 [3]	HADS (depression) Patient questionnaire (COPD symptoms time of day)	Experiencing symptoms in the morning, daytime, and nighttime was associated with anxiety and depression (*p* < 0.001)
Martinez Rivera et al. Lung 2016 [45]	HADS (depression) MRC scale (dyspnea)	Greater dyspnea in patients with depression
***Sleep***		
Partridge et al. Curr Med Res Opin. 2009 [17]	Patient questionnaire (COPD symptoms)	Patients experiencing general fatigue and tiredness reported worse nighttime symptoms (*p* = 0.003)
Kessler et al. Eur Respir J. 2011 [5]	Patient interview (sleep and COPD symptoms)	A quarter of the total study population reported that their COPD symptoms had affected sleep quality
Scharf et al. Int J Chron Obstruct Pulmon Dis. 2011 [51]	Pittsburgh Sleep Quality Index (sleep) Sleep Symptom Questionnaire (nighttime sleep symptoms of COPD)	Sleep time correlated with the number of nocturnal symptoms such as wheezing, worrying, and uncontrolled thoughts (*p* < 0.0001). Specific respiratory symptoms were not significantly associated with low sleep times
Omachi et al. Sleep Med. 2012 [52]	Medical Outcomes Study sleep battery (sleep) MRC scale (dyspnea) Patient interview (dyspnea and cough)	Patients with cough symptoms had three-fold greater likelihood of disturbed sleep (*p* = 0.034) The degree of dyspnea was associated with a higher likelihood of disturbed sleep (*p* = 0.004)
Price et al. Int J Chron Obstruct Pulmon Dis. 2013 [13]	Jenkins Sleep Questionnaire Seven-point Likert scale Frequency of nocturnal awakening	Patients with nighttime symptoms are significantly more likely to experience sleep disturbance vs those without nighttime symptoms (*p* < 0.0001)
Miravitlles et al. Respir Res. 2014 [3]	COPD and Asthma Sleep Impact Scale (sleep quality) Symptom questionnaire (COPD symptoms time of day)	Experiencing symptoms in the morning, daytime, and nighttime was associated with sleep impairment (*p* < 0.001)
***Exacerbations and disease prognosis***		
Nishimura et al. Chest 2002 [54]	Modified 5-point grading scale (dyspnea) 5-year cumulative survival rate (mortality)	The level of dyspnea was associated with a lower 5-year survival rate (*p* < 0.001)
Burgel et al. Chest 2009 [56]	Patient questionnaire (COPD symptoms and exacerbations) Medical records (exacerbations)	Productive cough was independently associated with frequent exacerbations (≥2 in the previous year) (*p* < 0.0001)
Lange et al. Eur Respir J. 2014 [21]	Patient questionnaire (COPD symptoms) Hospital admissions data (previous and follow-up exacerbations)	Patients with nighttime dyspnea were more likely to have had ≥2 exacerbations in the previous year (*p* < 0.001). Nighttime symptoms were associated with future exacerbations (HR 2.3; 95% CI 1.7, 3.0)
Putcha et al. COPD 2014 [58]	Patient questionnaire (COPD symptoms) Mortality data (mortality)	Cough and phlegm symptoms together were associated with an increased risk of mortality (HR 1.27; 95% CI 1.02, 1.59)
Lindberg et al. Respir Med. 2015 [57]	Patient interview (COPD symptoms and exacerbations) Mortality data (mortality)	Patients with a productive cough have an increased risk of exacerbations (OR 9.25; 95% CI 6.23, 13.75), and a significantly increased risk of mortality (HR 1.48; 95% CI 1.13, 1.94)
Miravitlles et al. COPD 2016 [25]	Patient questionnaire (COPD symptoms) Hospital admissions data (follow-up exacerbations)	Early-morning and daytime symptoms were associated with exacerbations during follow-up (both *p* < 0.01), however significance was not maintained when adjusted for potential confounding factors

*6MWT* 6-Min Walk Test, *CAT* COPD Assessment Test, *CI* confidence interval, *COPD* chronic obstructive pulmonary disease, *EQ-5D* EuroQol five dimensions questionnaire, *HADS* Hospital Anxiety and Depression Scale, *HR* hazard ratio, *HRQoL* health-related quality of life, *mMRC* modified Medical Research Council, *OR* odds ratio, *PCP* primary care physician, *QoL* quality of life, *SGRQ* St. George’s Respiratory Questionnaire, *UCSD* University of California San Diego

## Abbreviations

6MWT: 6-Min Walk Test
CAT: COPD Assessment Test
CI: Confidence interval
COPD: Chronic obstructive pulmonary disease
DEPREPOC: Depression in chronic obstructive pulmonary disease
EQ-5D: EuroQol five dimensions questionnaire
FEV_1_: Forced expiratory volume in 1 s
GOLD: Global initiative for chronic Obstructive Lung Disease
HADS: Hospital Anxiety and Depression Scale
HR: Hazard ratio
HRQoL: Health-related quality of life
mMRC: modified Medical Research Council
n.r.: Not reported
OR: Odds ratio
PCP: Primary care physician
QoL: Quality of life
SGRQ: St George’s Respiratory Questionnaire
UCSD: University of California San Diego
